# Reproductive Success and Diet of the Swainson’s Hawk (*Buteo swainsoni*) in the Grasslands of Janos, Chihuahua, Mexico

**DOI:** 10.3390/ani16010131

**Published:** 2026-01-02

**Authors:** Nereyda N. Cruz Maldonado, Cayetano J. Villareal Lozoya, Javier Cruz Nieto, Alina Olalla Kerstupp, Gabriel Ruiz Aymá, Antonio Guzmán Velasco, José I. González Rojas

**Affiliations:** 1 Comisión Nacional Forestal (CONAFOR), Pasaje Siglo XXI N° 203, Fracc. El Sauz, Saltillo 25294, Coahuila, Mexico; nerecruz@gmail.com; 2 World Wildlife Fund (WWF), Ave. Insurgentes Sur 1216, Tlacoquemecatl del Valle, Benito Juárez, Ciudad de México 03100, Mexico; jvillareal@wwfmex.org; 3 Organización Vida Silvestre, A.C. (OVIS), Keramos Pte 225, Col. Del Prado, Monterrey 64410, Nuevo León, Mexico; j.cruz@ovis.org.mx; 4 Universidad Autónoma de Nuevo León (UANL), Facultad de Ciencias Biológicas, Laboratorio de Biología de la Conservación y Desarrollo Sustentable, Cd. Universitaria, San Nicolás de los Garza 66455, Nuevo León, Mexico; alina.olallakrs@uanl.edu.mx (A.O.K.); gabriel.ruizym@uanl.edu.mx (G.R.A.); antonio.guzmanvl@uanl.edu.mx (A.G.V.); 5 Universidad Autónoma de Nuevo León (UANL), Facultad de Ciencias Biológicas, Laboratorio de Ornitología, Cd. Universitaria, San Nicolás de los Garza 66455, Nuevo León, Mexico

**Keywords:** Swainson’s Hawk, nesting ecology, reproductive success, diet, predation, grasslands, México

## Abstract

Understanding the breeding ecology and trophic dynamics of the Swainson’s Hawk (*Buteo swainsoni*) is essential for conserving grassland raptor populations in northern Mexico. We evaluated reproductive success, nest-site characteristics, and diet of the species in the grasslands of Janos, Chihuahua, during the 2006 breeding season. Eighteen nests were monitored to estimate daily survival rates (DSRs) using the Mayfield method. Overall nest success was 44.4%. DSR declined significantly from incubation (0.99 ± 0.00079) to the nestling stage (0.98 ± 0.00087; *z* = 8.5, *p* < 0.001), resulting in cumulative survival of 79.9% and 56.2%, respectively. Successful nests tended to occur farther from towns, although this trend was not statistically significant. Most nests were built in mesquite trees at intermediate elevations and in areas with low human disturbance. Diet analyses of 56 pellets and 91 prey remains revealed a predominance of vertebrates (63.17%), mainly mammals and reptiles, with vertebrate frequency significantly exceeding that of invertebrates (χ^2^ = 23.19, *p* < 0.001). These results highlight the species’ reliance on vertebrate prey and the vulnerability of the nestling stage, underscoring the importance of long-term monitoring in semi-arid grasslands.

## Simple Summary

The Swainson’s Hawk is a migratory raptor that breeds in the grasslands of northern Mexico, but little is known about its ecology in this region. We studied its reproduction, nest locations, and diet in the Janos grasslands of Chihuahua. We monitored 18 nests and found that less than half produced fledglings, mainly because young hawks (nestlings) had much lower survival than eggs during incubation. Nests were usually placed in mesquite trees located away from towns and in areas with low human disturbance. By analyzing pellets and prey remains, we found that the hawks fed mostly on vertebrates, such as small mammals and lizards. These findings highlight the importance of conserving natural grasslands and monitoring populations over time to support this species’ long-term survival.

## 1. Introduction

The grasslands of the Chihuahuan Desert are critical areas for nesting, migration, and winter habitat for numerous species of high conservation interest in the United States. In Mexico, the grasslands of the Janos region in Chihuahua serve as a crucial ecosystem for preserving biodiversity in North America [1,2,3,4]. The study of the Swainson’s Hawk (*Buteo swainsoni*) in northern Mexico is of great importance, as this region constitutes a key area for its reproduction and migration, allowing the evaluation of aspects of its ecology, productivity, and diet in a priority ecosystem for conservation, such as the grasslands of Janos, Chihuahua. Understanding its population dynamics in these habitats contributes not only to the scientific knowledge of the species but also to the conservation of migratory raptors and the ecosystems that sustain their life cycle [5]. This species is considered in Mexico as “subject to special protection” under PROY-NOM-059-SEMARNAT-2025 [6].

The Swainson’s Hawk nests in southern Canada, western United States, and northern Mexico [7,8,9,10], with documented presence in the Mexican states of Chihuahua, Coahuila, Durango, Nuevo León, Tamaulipas, and Sonora [9]. This species migrates to South America, primarily Argentina, where it spends the winter [10]. Its typical habitat consists of open grasslands characterized by relatively sparse vegetation and few shrubs [10,11,12,13,14].

Regarding reproductive habits, the Swainson’s Hawk is a monogamous species that typically returns to the same breeding territories each year. Nesting usually occurs in open grasslands or agricultural areas, where pairs build large stick nests in isolated trees, shrubs, or occasionally on artificial structures. The female lays two to four eggs, which are mainly incubated by her, while the male provides food [10]. Nesting success rates have been documented in Canada [15,16] and the USA (Colorado [17,18], North and South Dakota [19,20], New Mexico [21], Wyoming [22], and California [23]).

The species primarily exhibits diurnal and opportunistic hunting habits. Its hunting strategy combines circling flights to detect prey at a distance with rapid dives from perches or low flights over the ground. It feeds on a wide variety of prey, showing flexibility in its diet depending on resource availability [17], consisting mainly of mammals, followed by birds, and to a lesser extent, herpetofauna. In addition, the consumption of insects from various orders and families has been documented [10,11,18,19,21,22,24,25,26,27,28].

In Mexico, particularly in the North of the country, studies on this species are scarce. Two investigations have been conducted in the Mapimí Biosphere Reserve in Durango, focusing on reproductive success, nest site characteristics, diet, territoriality, and competition [27,29]. Additionally, two studies in the Janos region, Chihuahua, reported the presence of 17 Swainson’s Hawks [30] and the consumption of Texas horned lizards (*Phrynosoma cornutum*) as prey [31].

The present study aims to contribute to the knowledge of the ecology of the Swainson’s Hawk in the grasslands of Janos, Chihuahua, focusing on its reproductive success, nest site characterization, and diet. We hypothesize that the reproductive success of *Buteo swainsoni* is primarily driven by the degree of anthropogenic disturbance, by natural variation in survival throughout the breeding cycle, and by the composition of the diet during the breeding season. We predict that successful nests will occur in less-disturbed areas located farther from human settlements; that daily survival rates will be higher during incubation and will decline as nestlings and fledglings develop, reflecting a gradual increase in cumulative mortality; and that the breeding-season diet will be dominated by vertebrate prey, exhibiting a higher frequency of occurrence than invertebrates.

## 2. Materials and Methods

### 2.1. Study Area

The study was conducted at Rancho El Uno, located in the municipality of Janos, Chihuahua. This area is situated in the northwestern region of the state, just south of the United States border and to the east of the state of Sonora, Mexico. Its geographic coordinates are latitude 30°50′54″ N and longitude 108°32′18″ W at an elevation of 1415 m above sea level. It is part of the Janos Valley, where four ecoregions converge: the Chihuahuan Desert, the Sonoran Desert, the Sierra Madre Occidental, and the Apachería Region. The climate is temperate and arid. The average annual temperature is 15.7 °C, with an average of 6.0 °C in January and 26.1 °C in June. Annual precipitation is 381 mm, and 77 percent of the rain falls between April and August [32]. The vegetation forms a mosaic of natural and modified areas [33], with three main types predominating: grasslands, scrublands, and forests. The fauna is diverse, with a total of 383 terrestrial vertebrate species. Birds are the best-represented group, with 257 species, while amphibians are the least numerous, with 13 species [34].

### 2.2. Location and Monitoring of Nests

Nest searches were conducted during May 2006. Opportunistic observations were made by vehicle on roads and on foot, when necessary, between the hours of 6:00 a.m. and 11:00 a.m. and 5:00 p.m. and 7:00 p.m. Nests were identified with binoculars and georeferenced using a GPS. Once identified, the nests were monitored at variable intervals—ranging from 5 to 16 days—from May to August. To minimize disturbance, binoculars and a telescope Leica APO Televid 770 from Leica Camera AG (Wetzlar, Germany) were used to observe the nests from a safe distance (150–200 m) [35]. In the brief moments when the nest was left unsupervised by the parents, the nest was internally inspected to determine the number of eggs laid and the number of hatched chicks using a telescopic inspection mirror from Forestry Suppliers, Inc. (Jackson, MS, USA), that extends to 17.5 feet for maximum reach. All procedures followed low-disturbance protocols recommended for raptor nest monitoring [36].

### 2.3. Reproductive Success

In avian studies, reproductive success refers to the ability of an individual to produce offspring that survive to independence, commonly quantified as nest success (the proportion of nests that produce at least one fledgling) or fledging success (the number of young that leave the nest) [37]. Reproductive success in this study was estimated using the Mayfield method [38,39], which calculates the daily survival rate (DSR) of nests based on the total number of exposure days during which each nest remained active and, therefore, at risk of failure. This approach provides a more accurate estimate of reproductive success because it accounts for the fact that nests are often discovered at different stages of the breeding cycle. Rather than relying solely on the proportion of successful nests, the method estimates daily survival and extrapolates it to the entire nesting period, thereby reducing bias associated with unequal observation times.

Johnson’s statistical correction [40] was applied to minimize potential overestimation of reproductive success. From the estimated DSR, we derived daily mortality rates and cumulative survival probabilities for each reproductive stage, using average stage durations of 35 days for incubation and 44 days for fledglings [17,41,42,43]. Overall nest survival for the entire reproductive cycle was calculated by multiplying the stage-specific survival probabilities. A nest was considered successful when at least one fledgling was observed outside the nest. Mortality factors associated with each failure event were also recorded.

To evaluate whether successful nests were located farther from human settlements than failed nests, we compared distances to towns (>100 inhabitants) and ranches (<100 inhabitants) between both groups using Student’s *t*-tests. The relationship between the gradient of anthropogenic disturbance and nest success was further assessed using principal component (PCA) scores that described low-disturbance conditions. Correlations were calculated between nest success (0 = failed; 1 = successful) and these PCA components such that positive associations indicated higher success in less disturbed environments.

Daily survival rates were also compared among reproductive stages to test the prediction that survival decreases during later stages of the cycle. Stage-specific DSR values (in logit scale) were contrasted using *Z*-tests based on differences between estimates and their standard errors. The cumulative mortality for each stage was calculated from the corresponding DSR values.

### 2.4. Nest Site Characteristics

At the end of the breeding season, we recorded the structural and spatial characteristics of nest sites. For each nest, we identified the tree species, measured tree height and nest height using a laser rangefinder (Bushnell Elite 1 Mile), and recorded trunk diameter at chest height (DCH) with a Forestry Suppliers metric diameter tape. The treetop area (horizontal canopy projection) was calculated following standard forestry procedures [44] by measuring the longest (*a*) and perpendicular (*b*) crown diameters and estimating the ellipse area as *A* = π × *a* × *b*/4. Nest dimensions (height, width, diameter, and depth) were measured with a Forestry Suppliers metric diameter tape.

Geographic coordinates of each nest were processed in a GIS to determine elevation above sea level and to quantify Euclidean distances to the nearest Swainson’s Hawk nest, permanent water sources, unpaved rural roads, small ranches, nearby towns, and agricultural fields. We performed a principal component analysis (PCA) to identify the main environmental and spatial gradients associated with nest-site selection [42]. All variables were standardized (mean = 0, SD = 1) prior to analysis. The PCA was conducted in R (version 4.4.0) using the FactoMineR and factoextra packages and cross-validated in Python using scikit-learn (version 1.5). Additionally, we calculated Spearman’s correlation coefficients between nest success (1 = successful; 0 = failed) and each environmental variable [45].

### 2.5. Diet Determination

During June, July, and August, regurgitated pellets and prey remains were collected by manually accessing nests with a ladder. To minimize disturbance, samples were collected once per month per nest only when adults were absent. All material was stored individually in plastic jars and paper bags for subsequent prey identification.

Mammalian prey were identified using skulls, teeth, and hair [46,47]; herpetofauna were identified using scales, skulls, and other diagnostic skeletal elements [48,49]; birds were identified using beaks, skulls, bones, feathers, and available taxonomic descriptions and illustrations [9]. Invertebrates were identified based on diagnostic exoskeletal structures [50]. Taxonomic identification was complemented by comparisons with reference specimens from the mammalogy, herpetology, ornithology, and entomology collections of the Faculty of Biological Sciences, Universidad Autónoma de Nuevo León. Diet composition was described using the relative frequency of occurrence of each prey category [51].

To test the prediction that vertebrates dominated the breeding-season diet, we compared the total frequency of vertebrate versus invertebrate prey using an χ^2^ test. Ninety-five percent confidence intervals for vertebrate and invertebrate proportions were calculated using Wilson’s method.

## 3. Results

### 3.1. Reproductive Success

All nests were found in the incubation stage. The nesting period, from incubation to the fledgling stage, was recorded from early May to early August 2006, with an average duration of 78 days (n = 18 nests). Incubation lasted 35 days, while the parental care period extended for 44 days.

To estimate the start of egg-laying, a 35-day countdown was conducted from the hatching date of the first chick, following the methodology of Kaufman [43]. Based on this, the egg-laying period occurred between 6 May and 18 May, hatching took place between 10 June and 22 June, and fledgling emergence occurred from 26 July to 8 August. The productivity rates obtained for the Swainson’s Hawk were as follows: 45% of nests were successful, with 75% hatching success per nest and 47.62% fledging success per chick, resulting in an overall survival rate of 35.71% (Table 1).

Daily survival rates differed significantly between stages. During incubation, DSR was 0.99 ± 0.00079, yielding a cumulative survival of 79.9%. In contrast, nestling-stage DSR decreased to 0.98 ± 0.00087, a highly significant reduction (z = 8.5, *p* < 0.001), resulting in a cumulative survival probability of 56.2%. Consequently, the overall survival probability across the entire reproductive cycle was 44.8%, indicating that the nestling stage represents the most vulnerable phase (Table 2).

The leading cause of mortality during the incubation period was predation (14.28%), followed by broken eggs (7.14%) and unhatched eggs (3.57%). During the nestling stage, predation was also the most significant cause (23.80%), followed by nestlings falling from the nest (14.28%), nest abandonment (4.76%), and nestlings entangled in artificial materials (4.76%).

### 3.2. Nest Site Characteristics

A total of 18 nests were recorded, of which 89% were in mesquite trees (*Prosopis glandulosa*) and 11% were in spiny hackberry (*Celtis laevigata*) (Table 3). The nests were composed of three layers:

1. A main structure made of dry mesquite branches.
2. An intermediate layer consisting of grass, fresh mesquite leaves, and small green willow (*Salix* sp.) branches.
3. A top layer that included artificial materials such as plastic raffia and bags

**Figure 1:**
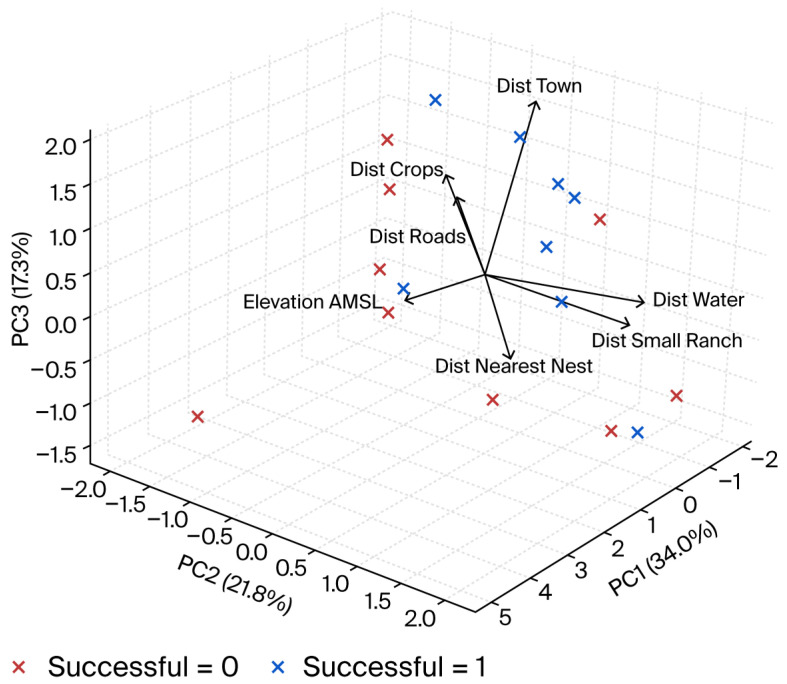
Three-dimensional PCA plot of *Buteo swainsoni* nests. Axes (PC1–PC3) explain 73% variance. Red = unsuccessful, blue = successful.

The principal component analysis revealed that three factors account for 73% of the total variance. The first factor showed a strong correlation with the nests, primarily related to elevation (mamsl), distance to the nearest nest, and proximity to agricultural fields. These results indicate that Swainson’s Hawks tend to build their nests at intermediate elevations, between 1398 and 1478 mamsl., in areas that are relatively homogeneous in topography, spaced apart from each other, and located far from cultivated zones. The second factor showed a significant correlation between the distance to water and distance to localities or ranches; this could reflect a preference for less disturbed areas, with low human activity and a more stable environment for nesting. The third factor was associated with the distance to towns, indicating that nests tend to be far from urban areas or densely populated localities (Table 4 and Table 5 and Figure 1).

To evaluate whether reproductive success was associated with human disturbance, we compared distances from nests to anthropogenic features. Although not statistically significant (t = −2.04, df = 16, *p* = 0.05), successful nests tended to be located farther from towns (>100 inhabitants) than unsuccessful ones. No differences were detected in distances to ranches, roads, water sources, or agricultural fields (*p* > 0.05). These patterns suggest a trend toward higher success in less disturbed areas, although statistical support is inconclusive.

### 3.3. Diet Composition

A total of 56 pellets and 91 remnants were collected and analyzed (remnants are remains of prey that were NOT ingested), with 71.55% of the items identified and 28.45% consisting of unidentified material. The diet of the Swainson’s Hawk is composed mainly of vertebrates (63.15%) and invertebrates (36.85%) (Table 6).

Among the vertebrates, mammals were the most prominent group, representing 36.54% of the diet. Within this group, the order Rodentia was dominant (50%), followed by Lagomorpha (22.14%). The families present in the diet included Heteromyidae (22.93%), Leporidae (16.39%), and Geomyidae (12.28%). The second most frequent group was herpetofauna (20.36%), primarily represented by the family Phrynosomatidae (95.57%), and then followed by birds (6.29%). The main prey species of the Swainson’s Hawk were the Texas horned lizard (*Phrynosoma cornutum*), banner-tailed kangaroo rat (*Dipodomys spectabilis*), black-tailed jackrabbit (*Lepus californicus*), and Botta’s pocket gopher (*Thomomys bottae*) (Table 6).

The most common invertebrate orders found in this raptor’s diet were Hymenoptera (40.64%) and Coleoptera (39.01%), with Formicidae (36.57%) and Scarabaeidae (8.93%) being the most notable (Table 6).

Formal statistical tests supported the dominance of vertebrates in the diet: Vertebrate occurrence (0.63, 95% CI: 57.9–68.2) was significantly higher than invertebrate occurrence (χ^2^ = 23.19, df = 1, *p* < 0.001). No differences were detected when considering pellets alone (χ^2^ = 0.04, df = 1, *p* = 0.85), whereas prey remains showed a strong vertebrate bias (vertebrate proportion = 0.97, 95% CI: 90.8–98.9; χ^2^ = 79.40, df = 1, *p* < 0.001), consistent with detectability biases favoring vertebrate prey.

## 4. Discussion

### 4.1. Nest-Site Selection

Most Swainson’s Hawk nests were found in mesquite trees, which aligns with the findings of Rodríguez-Estrella [29] and Nishida et al. [52], who highlight that mesquite provides both perching sites and habitat for prey species [53]. The average height of the trees (4.16 m) and the nests (3.05 m) fall within the range documented for this species in various previous studies [19,27,29,52]. Nest dimensions were similar to those reported previously [54,55]. Regarding construction materials, our findings align with Fitzner’s [56] observations, which described nests as primarily composed of small, fresh branches, grasses, and occasionally anthropogenic materials, such as wire, rope, or farming tools.

Elevation emerged as the main contributor to the first principal component in the PCA (loading = 0.96), indicating that variation in nest-site distribution was primarily associated with altitudinal gradients. Although the study area presents limited topographic relief (1398–1478 mamsl), this pattern suggests that Swainson’s Hawks select nesting sites located at intermediate elevations within relatively homogeneous terrain. Such areas likely offer favorable visibility for detecting predators and prey, reduced risk of flooding, and proximity to open grasslands suitable for hunting. Similar relationships between elevation and nest-site selection have been reported for other *Buteo* species, where moderate elevations provide optimal combinations of vegetation structure, prey availability, and microclimatic stability [57,58,59,60]. Consequently, elevation in this context reflects not steep slopes but the overall landscape position that provides both structural stability for nests and access to suitable foraging areas.

The second principal component was mainly associated with distance to water (loading = 0.80), indicating that this environmental gradient contributes to the differentiation of nest-site locations. Positive loading suggests that nests with higher PC2 scores are located farther from permanent water sources. In semi-arid grasslands such as those in Janos, water bodies often concentrate livestock and human activity, potentially increasing disturbance and competition for suitable nesting trees [59]. Conversely, areas located farther from water tend to have more open vegetation and reduced anthropogenic presence, conditions that enhance hunting efficiency and nest protection [58,61]. Similar patterns have been observed in other *Buteo* species occupying arid environments, where nesting at moderate or greater distances from water provides a balance between prey availability and reduced risk of human disturbance [54,62]. This result suggests that Swainson’s Hawks in Janos may favor nesting areas located at intermediate to greater distances from water sources, optimizing both foraging opportunities and reproductive safety.

Importantly, although most spatial patterns were consistent with environmental gradients, the statistical comparison of successful versus failed nests only revealed a non-significant trend toward successful nests being located farther from towns (>100 inhabitants). This aligns with the PCA indication that distance to towns contributes to nest-site differentiation, although the statistical evidence is not conclusive. Thus, nesting farther from dense human settlements may provide certain advantages, but these effects appear subtle in the short term.

In general terms, principal component analysis indicates that, in Janos, Swainson’s Hawks select nesting sites characterized by specific environmental and spatial features. Among the strongly associated variables is the choice of sites far from human pressure and activity, such as cultivated landscapes and densely populated localities and towns. Similarly, Groskorth [63] reported that nests in Canada were associated with greater grassland, tree, and shrub cover and with less cultivated land.

This pattern contrasts with what was reported by Nishida et al. [52] in southeastern Arizona, where the species showed higher nesting densities in agricultural areas than in grasslands or desert scrub, but interestingly, reproductive success and productivity did not differ significantly between land-cover types. The species nested in planted shelterbelts or orchard trees in agricultural landscapes and mesquite in natural habitats, indicating that nest-site selection was driven primarily by tree height and vegetative cover rather than by the surrounding matrix. This study did not provide evidence that hawks were forced to nest in agricultural areas due to a lack of natural sites; instead, both agricultural plantings and woody encroachment in grasslands have increased the availability of suitable nest trees, suggesting that Swainson’s Hawks adapt well to altered landscapes if adequate nest structures are present.

Similarly to Nishida et al. [52], Inselman et al. [20] and Dechant et al. [64] noted that the species can tolerate certain levels of disturbance if various structures are available for nesting, and it may even persist in landscapes with agricultural and farm use if trees or open areas for foraging are available. In Janos, nests were primarily located away from agricultural zones, possibly reflecting regional differences in tree availability, crop type, or anthropogenic pressure. This pattern is consistent with the findings of Rodríguez-Estrella [29] and Restani [65], who observed similar behaviors in the Mapimí Biosphere Reserve and Montana, respectively. Restani [65] also found that nests were distributed in an aggregated manner, resulting in shorter distances between nests than those reported for Janos.

Overall, these results suggest a degree of regional ecological plasticity in the nest-site selection of *Buteo swainsoni*, which appears to be mediated by landscape structure, degree of human disturbance, availability of suitable nesting trees, and local prey abundance. While this species may utilize agricultural and farm habitats in some regions when certain structural elements are preserved, in less transformed landscapes such as Janos, it tends to select more isolated sites with less human interference. This behavior could have important implications for its conservation at both local and regional scales.

### 4.2. Productivity

In the Janos region, an average of 1.56 eggs per nest was recorded, a value similar to the average of 2 eggs documented for northern Mexico [27,29]. In contrast, in various locations across the United States, values range between 2.2 and 2.5 eggs per nest [17,22,24,56]. The average of 0.56 fledglings per nest was lower than the reported range of 1.11 to 1.67 fledglings for this species. However, the average of 1.25 fledglings per successful nest falls within the documented range for other regions, which varies between 1.05 and 2.18 fledglings [17,18,19,20,21,22,24,29,54,56,66,67,68,69,70].

The percentage of successful nests in Janos was 44.4%, a value slightly higher than that reported by Inselman et al. (34% [20]) in North and South Dakota (USA), but it was lower than those reported in various regions of North America: Alberta (71.2%; [54]), Washington (81.3%; [56]), Colorado (54.6% [67]), Idaho (76.5% and 71.1%; [68,71]), California (65.5%; [70]), New Mexico (81%; [21]), and Durango, Mexico (75%; [29]). According to estimates from the Mayfield method, nesting success in Janos was estimated at 44.9%, a value within the range reported for southeastern Arizona (44–58% [52]) and close to the 48% documented for Colorado [18].

Importantly, stage-specific survival analysis showed that the nestling stage exhibited significantly lower daily survival rates than incubation, confirming that nestling vulnerability is a key limiting factor. Cumulative mortality in nestlings (43.8%) was more than double that of incubation (20.1%), emphasizing the demographic importance of this stage.

Inselman et al. [20] assume that their poor breeding success may be related to the late migratory behavior of this species, which requires them to occupy marginal habitat due to other raptors occupying the most suitable habitat before Swainson’s hawks arrive at the breeding grounds. Therefore, they have less access to prey. Schmutz et al. [15,16] mentioned that the declines in reproduction and survival of adult Swainson’s Hawks in Canada between 1972 and 2003 were a direct result of declines in the abundance of ground squirrels (their primary prey).

Our study did not determine prey availability, but due to the wide variety of prey found in both pellets and remnants, we believe that the low percentage of successful nests in Janos may be related to storms and strong winds recorded during the study period, which destroyed several nests, consistent with observations by Gilmer and Stewart [19]. Additionally, during the incubation and nestling stages, predation was the leading cause of mortality.

Although incubation appears to be relatively safe, the significantly lower DSR and higher cumulative mortality in the nestling stage show that this is the phase that most strongly limits recruitment. This underscores the importance of the long-term monitoring of post-fledging survival, since post-chick mortality substantially influences population dynamics [72]. Nishida [52] indicates that the hawk’s reproductive rate is known to fluctuate among years in response to environmental conditions; thus, it is advisable, as suggested by Briggs et al. [23], to conduct long-term reproductive monitoring, as short-term studies may not provide enough information to obtain reliable survival estimates, for example, for long-lived, widely distributed, or difficult-to-capture species such as the Swainson’s hawk.

These long-term monitoring measures should include not only observations of reproductive success but also long-term survival, reproductive recruitment rate, diet and prey availability, and stochastic events and anthropogenic pressures.

### 4.3. Diet

Diet composition was assessed from pellets and prey remains collected at 18 active nests during the breeding season. Although pellets and remains were collected at all nests, the number of samples obtained per nest was not recorded. Consequently, we could not standardize sampling effort among nests or evaluate within-nest variation in diet. Our results should therefore be interpreted as a population-level description of the breeding-season diet of Swainson’s Hawks in the study area, rather than as precise estimates of prey consumption per pair or as comparative diet data among nests. In addition, as with all pellet- and remains-based studies, the reconstructed diet is likely biased toward prey that produce identifiable hard parts (e.g., bones, feathers, scales), potentially underrepresenting soft-bodied prey.

The breeding diet of the breeding population of Swainson’s Hawk (*Buteo swainsoni*) in the Janos Biosphere Reserve is composed mainly of vertebrates (63.17%), which is consistent with findings from various authors [26,53,54,65,73]. Within this group, mammals account for 36.53% of the prey consumed, a proportion higher than the 15–22% recorded in the Mapimí Biosphere Reserve, Mexico [29], but lower than the 45–95% range reported in other studies [18,19,22,24,54,65].

The primary mammalian prey consumed by the Swainson’s Hawk in Janos was the banner-tailed kangaroo rat (*Dipodomys spectabilis*), with a frequency of 8.38%. In contrast, several studies have reported different species of pocket gophers as predominant prey, such as the northern pocket gopher (*Thomomys talpoides*), with frequencies between 40% and 75% [19,24], and the plains pocket gopher (*Geomys bursarius*) and yellow-faced pocket gopher (*Cratogeomys castanops*), with a frequency of 5.9% [26]. Likewise, the cottontail rabbit (*Sylvilagus audubonii*, 17%; [21]) and spotted ground squirrel (*Spermophilus spilosoma*, 7.87–38.9%) have been documented as important prey in other regions [29,53,65]. The black-tailed jackrabbit (*Lepus californicus*) was also an important component of the diet, with a frequency of 5.99%, consistent with Rodríguez-Estrella [29], who reported frequencies ranging from 4.08% to 7.22%.

Among reptiles, the Texas horned lizard (*Phrynosoma cornutum*) was the most consumed item in the Janos region, with a frequency of 19.36%. This species not only had a high representation in the area but has also been identified as a key prey in other studies, with proportions ranging from 2.80% to 13.69% [21,26,29,31,53], highlighting its importance as a recurrent trophic resource throughout the hawk’s range.

Birds were present in the Swainson’s Hawk diet with a frequency of 6.29%, a proportion lower than that reported by Dunkle [22] (25%) and Andersen [18] (50%) but higher than that recorded by Bednarz [21] (1.2%), who identified scaled quail (*Callipepla squamata*) and northern bobwhite (*Colinus virginianus*) as the most frequently consumed species. In the case of Janos, Chihuahua, the only bird species recorded was the burrowing owl (*Athene cunicularia*), consistent with Chipman [74], who identified this owl as a potential prey of the hawk.

Invertebrates also represent an important group in the Swainson’s Hawk diet, with a frequency of 36.83%. This value is comparable to the one reported by Rodríguez-Estrella [29], who documented proportions of 36.87% and 57.15% in different years, and those reported by Bednarz [21] (54.9%) and Giovanni [26] (28%). These data underscore the relevance of invertebrates as a complementary food resource during the breeding season. In Janos, the most consumed invertebrate orders were Hymenoptera (14.97%) and Coleoptera (14.37%). The above contrasts with Bednarz [21], who found that Orthoptera were the most frequent group in the diet (35%). Similarly, Rodríguez-Estrella [29] reported Coleoptera as the dominant group (30.03% and 27.41%), while Giovanni [26] recorded a frequency of 14.90% for grasshoppers. These differences suggest spatial variations in prey availability or selection, possibly influenced by habitat type, the seasonal abundance of arthropods, or the foraging strategies employed by the species.

## 5. Conclusions

Our findings provide valuable insights into the reproductive performance and trophic ecology of the Swainson’s Hawk (*Buteo swainsoni*) in the grasslands of Janos, highlighting the species’ ecological plasticity and adaptability to variable environmental conditions. Nest-site selection was primarily associated with mesquite trees located in less disturbed areas, and although statistical comparisons did not yield significant differences, we detected a non-significant trend for successful nests to occur farther from densely populated towns. This pattern suggests that lower levels of human disturbance may confer subtle reproductive advantages, even if the current evidence remains inconclusive.

Reproductive success in the study area fell within the expected range for the species; however, stage-specific analyses revealed a clear demographic bottleneck. Daily survival rates declined significantly from the incubation to the nestling stage, and cumulative mortality during the nestling phase was more than double that observed during incubation. These results confirm that the nestling stage represents the most vulnerable period of the breeding cycle and underscore the importance of considering stage-specific sources of mortality—such as storms, nestling falls, and predation—when evaluating regional reproductive performance and population viability.

Diet analyses indicated that Swainson’s Hawks relied primarily on terrestrial vertebrates, particularly small mammals and lizards, with vertebrate prey occurring significantly more frequently than invertebrates during the breeding season. While prey remains showed a strong vertebrate bias compared to pellets—highlighting inherent detectability biases between sampling methods—the inclusion of both vertebrates and invertebrates reflects a flexible and opportunistic foraging strategy that likely enhances persistence in semi-arid environments.

Taken together, these patterns indicate that Swainson’s Hawks in Janos maintain a balanced ecological strategy, combining habitat selectivity in relatively undisturbed landscapes with adaptability in diet and resource use. Although this study is limited to a single breeding season and a relatively small number of nests, it addresses a critical information gap, as data on the breeding ecology of this species in Mexico remain extremely scarce and long-term or large-scale datasets are currently unavailable. In this context, our results provide valuable baseline information on nesting characteristics, reproductive parameters, and trophic ecology, establishing an essential reference for future research. Continued long-term monitoring of reproductive dynamics, stage-specific survival, prey availability, and environmental pressures will be fundamental for improving our understanding of population trends and for informing effective conservation strategies for this migratory raptor in northern Mexico’s grassland ecosystems.

## Figures and Tables

**Table 1:** Productivity measures for the Swainson’s Hawk in the grasslands at El Uno Ranch, Janos, Chihuahua.

Productivity Measures	$\overset{–}{x}$ ± S.E.
Number of nests	18
Successful nests (%)	44.4
Number of eggs	28
Average number of eggs per nest	1.56 ± 0.10
Average number of chicks per nest	1.17 ± 0.17
Hatching success per egg (%)	75
Success of fledglings per chick (%)	47.62
Success of fledglings per egg (%)	35.71
Average number of fledglings per nest	0.56 ± 0.70
Average number of fledglings per successful nest *	1.25 ± 0.46
Chick mortality per nest (%)	52.38
Egg mortality per nest (%)	25

$\overset{–}{x}$ = mean; S.E. = standard error. * The nest is considered successful when it has fledglings.

**Table 2:** Reproductive success for the Swainson’s Hawk in the grasslands at El Uno Ranch, Janos, Chihuahua.

	Egg Laying	Fledglings
Daily survival rate ± 1.96 (S.E.)	0.99 ± 1.96 (0.00079)	0.98 ± 1.96 (0.00087)
Daily survival rate C.I. (95%)	0.988–0.992	0.978–0.982
Daily death rate	0.006	0.013
Total daily survival probability	0.7987	0.56
Total daily survival probability C.I. (95%)	0.797–0.800	0.558–0.562
Total probability of survival for each phase	79.87%	56.22%
Total probability of survival ± 1.96 (S.E.)/C.I. (95%)	44.90% ± 1.96 (0.028)/44.84–44.95	

S.E. = standard error; C.I. = Confidence Intervals.

**Table 3:** Values obtained for Swainson’s Hawk trees and nests at El Uno Ranch, Janos, Chihuahua.

**(** **a) Tree**					
**Species**	**n**	**Tree height** **(m)**	**Nest height** **(m)**	DCH * **(cm)**	**Treetop** **(m^2^)**
*Prosopis glandulosa*	16	4.17 ± 0.35	3.14 ± 0.24	14.63 ± 1.06	08.19 ± 0.92
*Celtis laevigata*	2	4.13 ± 0.37	2.50 ± 0.15	23.25 ± 1.75	13.14 ± 2.13
**(** **b) Nests**					
	**n**	**Height** **(cm)**	**Width** **(cm)**	**Length** **(cm)**	**Depth** **(cm)**
	18	38.20 ± 2.06	49.71 ± 2.47	62.92 ± 2.51	7 ± 0.33

Values are presented as mean ± standard error. * Diameter at chest height.

**Table 4:** Nesting site characterization of the Swainson’s Hawk at El Uno Ranch, Janos, Chihuahua.

Nests (n = 18)	Elevation (mamsl)	Distance to Nearest Nest (km)	Distance to Water (km)	Distance to Unpaved Roads (km)	Distance to Small Ranch < 99 Inhabitants (km)	Distance to Towns > 100 Inhabitants (km)	Distance to Crop Field (km)
Mean	1415.03	1.79	2.19	2.57	2.52	10.81	4.65
S.E.	5.03	0.31	0.26	0.22	0.29	0.36	0.44
Range	1398–1487	0.21–4.92	0.67–4.25	0.87–4.41	0.39–4.70	8.30–13.16	1.30–7.19

S.E. = Standard Error; mamsl = meters above mean sea level.

**Table 5:** Principal component analysis conducted on nesting site variables of the Swainson’s Hawk at El Uno Ranch, Janos, Chihuahua.

	Principal Components		
Variable	1	2	3
Elevation (mamsl)	0.96	−0.09	0.008
Distance to nearest nest	0.78	0.42	−0.15
Distance to water	−0.30	0.80	0.05
Distance to unpaved roads	−0.50	−0.35	0.24
Distance to localities or ranches (1–99 inhabitants)	−0.19	0.76	−0.05
Distance to towns (+100 inhabitants)	−0.21	0.17	0.88
Distance to crop fields	0.70	−0.009	0.60
Cumulative variance	33.90	55.78	73.07

**Table 6:** Percent frequency of occurrence (FOC) of prey items from Swainson’s Hawk pellets and remains collected at El Uno Ranch, Janos, Chihuahua.

	Pellets (N = 56)		Remnants (N = 91)		Total	
	n	%FOC	n	%FOC	n	%FOC
VERTEBRATES	123	50.62	88	96.70	211	63.17
Mammals	60	24.69	62	68.14	122	36.54
Rodentia						
Heteromyidae						
* Dipodomys spectabilis*	9	3.70	19	20.88	28	8.38
Cricetidae						
* Sigmodon fulviventer*	4	1.65			4	1.20
*Neotoma* sp. (Cricetidae) = Muridae			1	1.10	1	0.30
Geomyidae						
*Thomomys bottae*	6	2.47	9	9.89	15	4.49
Sciuridae						
*Spermophilus spilosoma*	5	2.06	2	2.20	7	2.10
*Cynomys ludovicianus*	2	0.82	4	4.40	6	1.80
Lagomorpha						
Leporidae						
* Lepus californicus*			20	21.98	20	5.99
* Sylvilagus audobonii*			7	7.69	7	2.10
Mammals Undefined	34	13.99			34	10.18
Reptiles	43	17.70	25	27.48	68	20.36
Squamata						
Phrynosomatidae						
* Phrynosoma cornutum*	43	17.70	22	24.18	65	19.46
Teiidae			1	1.10	1	0.30
Colubridae			1	1.10	1	0.30
Anura						
Bufonidae			1	1.10	1	0.30
Birds	20	8.23	1	1.10	21	6.29
Strigiformes						
Strigidae						
* Athene cunicularia*			1	1.10	1	0.30
Birds Undefined	20	8.23			20	5.99
INVERTEBRATES (Insecta)	120	49.38	3	3.30	123	36.83
Hymenoptera	50	20.57	0		50	14.97
Formicidae	45	18.52			45	13.47
Pompilidae	2	0.82			2	0.60
Colletidae	1	0.41			1	0.30
Apidae	1	0.41			1	0.30
Sphecidae	1	0.41			1	0.30
Coleoptera	48	19.75	0		48	14.37
Scarabeidae	11	4.53			11	3.29
Anthicidae	2	0.82			2	0.60
Carabidae	1	0.41			1	0.30
Histeridae	1	0.41			1	0.30
Coleopteran unidentified	33	13.58			33	9.88
Orthoptera	7	2.88	2	2.20	9	2.70
Acrididae	2	0.82	1	1.10	3	0.90
Tettigonidae			1	1.10	1	0.30
Orthopteran unidentified	5	2.06			5	1.50
Diptera	6	2.46	0		6	1.80
Culicidae	3	1.23			3	0.90
Asilidae	1	0.41			1	0.30
Scatopsidae	1	0.41			1	0.30
Dipteran unidentified	1	0.41			1	0.30
Hemiptera	2	0.82	0		2	0.60
Largidae	1	0.41			1	0.30
Hemipteran unidentified	1	0.41			1	0.30
Lepidoptera	3	1.23	0		3	0.90
Tineiidae	2	0.82			2	0.60
Lepidopteran unidentified	1	0.41			1	0.30
Dermaptera	2	0.82			2	0.60
Trychoptera	2	0.82			2	0.60
Diplopoda			1	1.10	1	0.30
Total vertebrates identified	69	28.40	88	96.70	157	47.00
Total vertebrates unidentified	54	22.22	0	0	54	16.15
Total invertebrates identified	79	32.50	3	3.30	82	24.55
Total invertebrates unidentified	41	16.88	0	0	41	12.30
Total items	243	100	91	100	334	100

## Data Availability

The original contributions presented in the study are included in the article. Further inquiries can be directed to the corresponding author.
